# Efficacy evaluation of urethral dragging-and-adhesion anastomosis in ileal orthotopic neobladder reconstruction following laparoscopic radical cystectomy: a dual-center retrospective study

**DOI:** 10.3389/fsurg.2026.1874547

**Published:** 2026-07-22

**Authors:** Lie Yu Xu, Xin Chang Zou, Qing Wen Luo, Hai Chao Chao, Zun Wei Zhu, Xiang Da Xu, Tao Zeng

**Affiliations:** 1 Department of Urology, Jiangxi Provincial People’s Hospital (The First Affiliated Hospital of Nanchang Medical College), Nanchang, China; 2 Jiangxi Medical College, Nanchang University, Nanchang, China; 3 The Second Affiliated Hospital, Jiangxi Medical College, Nanchang University, Nanchang, China; 4 Department of Urology, Second Affiliated Hospital of Nanchang University, Nanchang, China

**Keywords:** dragging-and-adhesion anastomosis, immune function, laparoscopic radical cystectomy, orthotopic neobladder, urodynamics

## Abstract

**Background:**

Urethra–neobladder anastomosis following laparoscopic radical cystectomy combined with an ileal orthotopic neobladder is a pivotal step that significantly influences postoperative urinary continence, urodynamic performance, and the risk of surgical complications.

**Purpose:**

This study aims to evaluate the effects of a modified urethral dragging-and-adhesion anastomosis technique on postoperative urodynamic parameters, urinary continence recovery, and immune function, with emphasis on its potential benefits in reducing surgical trauma, enhancing functional outcomes, and mitigating immune suppression.

**Patients and methods:**

A retrospective analysis was performed on 55 patients with bladder cancer who underwent laparoscopic radical cystectomy with ileal orthotopic neobladder reconstruction at Jiangxi Provincial People's Hospital (35 cases) and the Second Affiliated Hospital of Nanchang University (20 cases) between June 2016 and January 2021. Of these, 30 patients received urethral dragging-and-adhesion anastomosis (observation group), while 25 patients underwent conventional interrupted anastomosis (control group). The study compared operative time and intraoperative blood loss between the two groups and evaluated postoperative urodynamic parameters, including maximum bladder capacity, postvoid residual, maximum flow rate (Q-max), and average flow rate (Q-ave). In addition, this study evaluated the recovery of daytime and nighttime urinary control in patients 3 months, 1 year, and 2 years after the surgery, along with immune function indicators 1 week after the surgery, namely, the proportions of CD3+, CD4+, and CD8+ T cells and the CD4+/CD8+ ratio.

**Results:**

There were no significant differences in preoperative general data or immune-related parameters between the two groups. The observation group demonstrated significantly shorter operative times (279.80 ± 45.32 min vs. 382.60 ± 45.94 min, *p* < 0.01) and urethral anastomosis times (18.37 ± 7.05 min vs. 61.80 ± 11.35 min, *p* < 0.01) compared with the control group, in addition to reduced intraoperative blood loss (274.00 ± 85.48 mL vs. 336.80 ± 72.44 mL, *p* < 0.01). No statistically significant differences were observed in urodynamic parameters between the two groups at 3 months, 1 year, or 2 years postoperatively (*p* > 0.05). Regarding urinary continence recovery, no significant differences were observed between the observation and control groups in daytime and nighttime continence rates at any postoperative time point (*p* > 0.05). Immune function tests revealed that the observation group had significantly higher postoperative levels of CD3+ T cells (57.63 ± 3.41% vs. 53.68 ± 3.05%, *p* < 0.01), CD4+ T cells (28.43 ± 2.66% vs. 24.56 ± 2.53%, *p* < 0.01) and an increased CD4+/CD8+ ratio (0.98 ± 0.15 vs. 0.83 ± 0.09, *p* < 0.01) compared with the control group. No significant difference was observed in CD8+ T-cell levels between the two groups (29.30 ± 3.04% vs. 29.72 ± 1.43%, *p* > 0.05). In addition, both groups showed significant postoperative reductions in CD3+, CD4+, and CD8+ T-cell counts, and in the CD4+/CD8+ ratio, compared to preoperative levels (*p* < 0.01).

**Conclusion:**

Urethral dragging-and-adhesion anastomosis is a safe and effective modified technique for urethral reconstruction during laparoscopic radical cystectomy with an ileal orthotopic neobladder. It enables speedy surgical completion and urethral anastomosis while preserving urodynamic and urinary continence functions comparable to those of traditional methods. In addition, it may contribute to better preservation of patients' immune function.

## Introduction

Bladder cancer is the tenth most prevalent malignancy worldwide, accounting for nearly 200,000 deaths every year (1, 2). In Western populations, it represents the fourth most common cancer among men (3). The initial diagnosis typically depends on painless gross hematuria as the primary symptom and cystoscopic confirmation. Notably, 20%–30% of non-muscle-invasive bladder cancer cases eventually progress to muscle-invasive disease. Moreover, high-grade Ta/T1 tumors demonstrate a concerning 30%–50% 5-year muscle-invasive recurrence rate following standard therapy, substantially compromising clinical outcomes (4–6). Radical cystectomy with urinary diversion constitutes the gold-standard treatment for muscle-invasive bladder cancer (7). Ileal orthotopic neobladder (IONB) reconstruction has emerged as the preferred diversion method owing to its physiological voiding advantages (8, 9). Nevertheless, this complex procedure poses substantial technical challenges, with postoperative complication rates of 30%–60% that markedly impair quality of life (10), placing stringent demands on urethral reconstruction.

The integrity of the urethra–neobladder anastomosis critically determines postoperative continence and complication profiles (11). Conventional interrupted suture techniques present inherent drawbacks, including inconsistent anastomotic tension and ischemic risks (12, 13). Our novel urethral dragging-and-adhesion anastomosis technique utilizes a simplified support mechanism that achieves precise anatomical alignment between urethral and neobladder tissues, ensures even tension distribution, and optimally preserves urethral vascularity—collectively reducing anastomotic leakage and stricture rates (14).

This study systematically compares the comprehensive effects of the urethral dragging-and-adhesion anastomosis technique versus traditional interrupted suture methods, evaluating its impact on patients' urodynamic parameters, 24-h urinary continence rates, and immune function indicators.

## Patients and methods

### Patients

We conducted a retrospective analysis of eligible patients who underwent laparoscopic radical cystectomy (LRC) with ileal orthotopic neobladder reconstruction at Jiangxi Provincial People's Hospital and the Second Affiliated Hospital of Nanchang University between June 2016 and January 2021. The patients were stratified into two cohorts based on the anastomotic technique: the observation group (urethral dragging-and-adhesion anastomosis, *n* = 30) and the control group (traditional interrupted anastomosis, *n* = 25). Inclusion criteria comprised the following: (1) histologically confirmed bladder cancer, (2) intact urethral anatomy with a functional external sphincter, (3) negative intraoperative urethral margins, and (4) absence of significant bowel pathology. Exclusion criteria included the following: (1) urethral tumor involvement or multifocal carcinoma *in situ* on preoperative cystourethroscopy, (2) prior radical radiotherapy or systemic chemotherapy, or complicated urethral stricture with a loss of self-care capacity, (3) concurrent malignancies, and (4) severe hepatic/renal insufficiency or systemic comorbidities. The institutional review boards of both participating centers approved the study protocol. All participants provided written informed consent prior to enrollment. Comprehensive clinical data were collected for all pathologically confirmed cases that met these stringent selection criteria.

### Patient data collection

Baseline patient parameters—including age, tumor stage, pathological type, and tumor grade—were collected. Intraoperative observation indicators included operative time, urethral anastomosis time, and intraoperative blood loss. Postoperative parameters assessed at 3, 12, and 24 months included indwelling catheter duration, postvoid residual (PVR), maximum flow rate (Qmax), and maximum bladder capacity (MBC). In addition, immune-related indicators were measured 1 week postoperatively. The number and frequency of pad usage were recorded at 3, 12, and 24 months postoperatively.

## Surgical methods

### Laparoscopic radical cystectomy with ileal orthotopic neobladder reconstruction

General anesthesia was administered with the patient in the supine position, supported by a hip pad and positioned with the head lower than the feet at a 30° angle. The surgeon stood on the patient's right side. A standard five-port laparoscopic radical cystectomy was performed, beginning with the insertion of a Veress needle at the supraumbilical margin to establish pneumoperitoneum at a pressure of 12–15 mmHg. A 30° laparoscope was used for visualization, and 12-mm trocars were inserted under direct vision at the lateral edges of the left and right rectus abdominis muscles, with an additional 12-mm trocar placed at the supraumbilical margin. Two 5-mm trocars were positioned medially, approximately two fingerbreadths from the level of the left and right anterior superior iliac spines. Preoperatively, a 20-Fr urethral catheter was indwelled, and an intravesical instillation of 50 mL containing 2.0 g of gemcitabine was administered via the urethral catheter. The abdominal organs were examined, the bilateral ureters were dissected free from the bladder, and the bilateral lymph nodes were removed. The bladder and prostate walls were separated until the prostatic apex was incised after suturing the deep dorsal venous complex. Following thorough dissection of both sides of the bladder and prostate, the bladder was completely excised. A 45–55 cm segment of ileum was harvested from the ileocecal region, arranged extracorporeally in a “W” configuration, and sutured to form a pouch. Both ureters were then implanted into the appropriate posterior positions of the neobladder. An opening, approximately the diameter of a 20-Fr Foley catheter (matching the thickness of the catheter), was created at the lowest part of the neobladder to facilitate urethral anastomosis.

### Neobladder–urethral anastomosis

**Figure 1:**
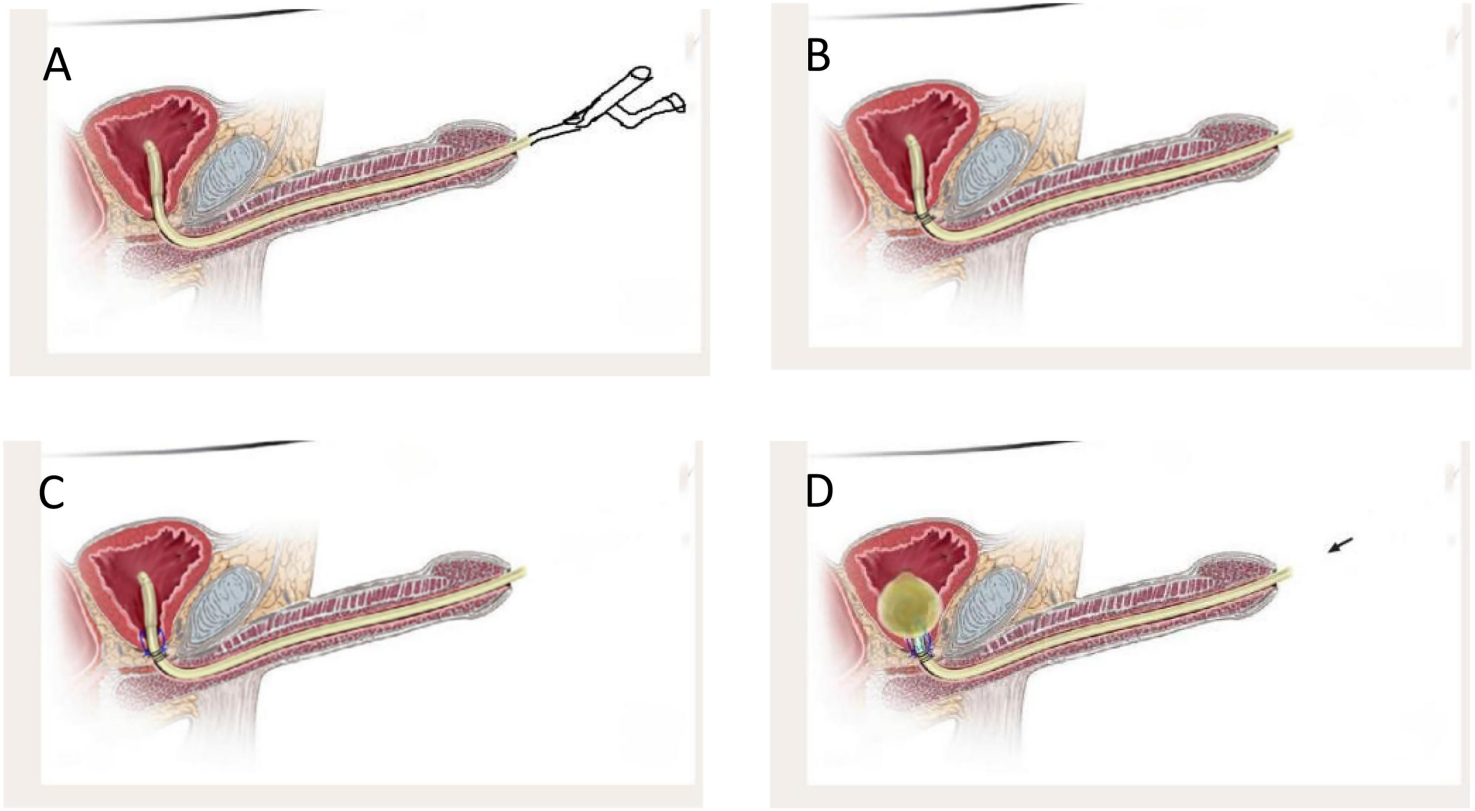
A schematic diagram of the urethral dragging-and-adhesion anastomosis technique used in bladder reconstruction. **(A)** Placement of a 20-Fr Foley catheter, **(B)** suturing and fixation around the catheter coil, **(C)** key location of the bladder–urethral anastomosis, and **(D)** inflating the catheter balloon to apply traction and achieve final fixation.

In the observation group, an anastomosis was performed laparoscopically using the urethral resistance and traction-assisted technique. First, a 20-Fr Foley catheter was inserted through the urethral orifice into the pelvic cavity. The catheter was then wrapped with 5–8 loops of 0–0.5 silk suture approximately 0.3–0.5 cm distal to the bladder catheter. Next, using 1-0 Vicryl sutures, the neobladder opening was anastomosed to the catheter loops at the 12, 2, 4, 6, 8, and 10 o'clock positions, creating a channel at the lowest part of the neobladder with a size matching that of the catheter. Finally, 15 mL of water was injected into the balloon, and the neobladder was positioned in the pelvis. The catheter was gently pulled outward and securely fixed at the external urethral orifice with moderate tension, facilitating a natural anastomosis through the traction effect of the catheter (Figure 1). In the control group, traditional interrupted sutures were used to perform the anastomosis under direct vision. The sutures were placed at the 6, 5, 7, 3, 9, and 12 o'clock positions. Each suture was initiated from the outer entrance and inner exit of the bladder neck and then continued from the inner entrance and outer exit of the urethra, ensuring that the knots remained on the outer side and that each entry point could be guided by the in-and-out movement of the catheter. The sutures were not tied until after the urinary catheter was positioned, with the final sutures and knots secured by the chief surgeon and assistant.

### Postoperative patient follow-up

Follow-up visits were conducted monthly for the first 3 months postoperatively, every 3 months from months 4 to 12, and every 6 months starting from the second year. Each follow-up visit included a urological color Doppler ultrasound, cystoscopy, and urethrography. At 3, 12, and 24 months postoperatively, urodynamic parameters were measured using a urodynamic analyzer, with primary indicators including maximum bladder capacity, residual urine volume, and maximum urinary flow rate. All tests were performed in accordance with the operational standards established by the International Continence Society (ICS). Before testing, patients were instructed to empty their bowels and fill their bladders. Initially, free urinary flow was measured with a uroflowmeter to determine the maximum flow rate. After bladder emptying, residual urine volume was assessed via catheterization. A double-lumen bladder pressure catheter was transurethrally inserted into the neobladder, and a rectal pressure catheter was placed in the anal canal. Physiological saline was infused into the bladder at a rate of 30–50 mL/min until the patient experienced urinary discomfort or reached maximum tolerance. Then, the maximum bladder capacity was recorded.

### Statistical analysis

The SPSS 27.0 software was used for all statistical analyses. Continuous data were expressed as the mean ± standard deviation, while categorical variables were presented as frequencies and percentages. A univariate analysis was performed to compare differences in clinical factors. The Student's *t*-test or Mann–Whitney *U* test was used for continuous variables, and the chi-square test or Fisher's exact test was employed for categorical variables. A two-sided *p*-value of less than 0.05 was considered statistically significant.

## Results

### Baseline patient characteristics

This retrospective study analyzed the clinical data of 55 male patients with invasive bladder cancer from two medical centers. Among these, 30 patients underwent neobladder–urethral dragging-and-adhesion anastomosis, while 25 patients received conventional interrupted neobladder–urethral anastomosis. The tumor characteristics of both groups are summarized in Table 1. There were no statistically significant differences between the two groups in terms of age, tumor T stage, pathological grade, or pathological type (all *p* > 0.05).

**Table 1:** General characteristics of the patients.

Characteristics	Observation group (*n* = 30)	Control group (*n* = 25)	*p*
Age (years)	70.10 ± 6.51	71.44 ± 6.21	0.44
Pathological stage			0.73
Tis	2 (7%)	1 (4%)	
T1	10 (33%)	7 (28%)	
T2	10 (33%)	12 (48%)	
T3	8 (27)	5 (20%)	
Tumor grade			0.93
G1	8 (27%)	6 (24%)	
G2	14 (46%)	13 (52)	
G3	8 (27%)	6 (24%)	
Pathological type [*n* (%)]			0.53
Transitional cell carcinoma	23 (77)	20 (80)	
Squamous carcinoma	5 (17)	2 (8)	
Glandular carcinoma	2(7)	3(12)	

### Perioperative data

All laparoscopic procedures were successfully completed without conversion to open surgeries. During follow-up, the mortality rates were comparable between the two groups, with two and five deaths occurring in the observation group at 1 and 2 years, respectively, versus two and six deaths in the control group. The observation group demonstrated significantly shorter operative times (279.80 ± 45.32  min vs. 382.6 ± 45.94 min, *p* < 0.01) and neobladder–urethral anastomosis times (18.37 ± 7.05  min vs. 61.80 ± 11.35 min，*p* < 0.01), along with reduced intraoperative blood loss (274.00 ± 85.48  mL vs. 336.80 ± 72.44 mL, *p* < 0.01) (Table 2). No significant differences were observed in urodynamic parameters at 3, 12, or 24 months postoperatively (all *p* > 0.05) nor in daytime or nighttime continence rates at any time point (*p* > 0.05), although both groups showed progressive improvement in maximum flow rate, average flow rate, and maximum bladder capacity over time (*p* < 0.01) (Table 3).

**Table 2:** Perioperative data.

Characteristics	Observation group (*n* = 30)	Control group (*n* = 25)	*p*
Surgery time (min)	279.8 ± 45.32	382.6 ± 45.94	<0.01
Urethral anastomosis time (min)	18.37 ± 7.05	61.80 ± 11.35	<0.01
Blood loss during surgery (mL)	274 ± 85.48	336.8 ± 72.44	<0.01
Catheter removal time (day)	17.20 ± 3.92	18.24 ± 2.92	0.28
Intestinal gas passage time (h)	38.87 ± 9.0	37.76 ± 10.46	0.67
Perioperative complications [*n* (%)]			0.71
Infection	5 (17)	3 (12)	
Gastrointestinal-related complications	3 (10)	2 (8)	
Urinary fistula	2 (6)	4 (16)	
None	20 (67)	16 (64)	

**Table 3:** Postoperative urinary control and urodynamic parameters.

Parameter	Time point	Observation group (*n* = 30)	Control group (*n* = 25)
Number of diapers (*n*)			
	3 months postoperatively		
	0–1	13	10
	≥2	17	15
	1 year postoperatively		
	0–1	16	12
	≥2	12	11
	2 years postoperatively		
	0–1	17	14
	≥2	8	5
Urination pad frequency (*n*)			
	3 months postoperatively		
	Day	14	12
	Night	16	13
	Day and night	0	0
	1 year postoperatively		
	Day	15	14
	Night	12	9
	Day and night	1	0
	2 years postoperatively		
	Day	18	14
	Night	4	2
	Day and night	3	3
PVR (mL)	3 months postoperatively	44.67 ± 11.96	41.20 ± 12.61
	1 year postoperatively	42.14 ± 9.47	40.39 ± 11.11
	2 years postoperatively	39.20 ± 11.24	38.58 ± 13.19
Q-max (mL/s)	3 months postoperatively	11.07 ± 2.24	10.36 ± 2.18
	1 year postoperatively	12.39 ± 2.13^a^	13.22 ± 2.25^a^
	2 years postoperatively	17.16 ± 3.93^b^	17.42 ± 4.61^b^
Q-ave (mL/s)	3 months postoperatively	7.17 ± 1.97	7.48 ± 2.08
	1 year postoperatively	9.82 ± 2.04^a^	10.70 ± 1.99^a^
	2 years postoperatively	13.76 ± 3.31^b^	14.53 ± 4.43^b^
MBC (mL)	3 months postoperatively	427.7 ± 29.67	438.80 ± 47.02
	1 year postoperatively	480.7 ± 34.85^a^	484.3 ± 32.17^a^
	2 years postoperatively	485.8 ± 41.47^b^	501.6 ± 41.80^b^

Data are presented as the mean ± standard deviation or the number of patients.

a Compared with the value at 3 months postoperatively, *p* < 0.05.

b Compared with the value at 1 year postoperatively, *p* < 0.05.

PVR, postvoid residual; Q-max, maximum flow rate; Q-ave, average flow rate; MBC, maximum bladder capacity.

**Figure 2:**
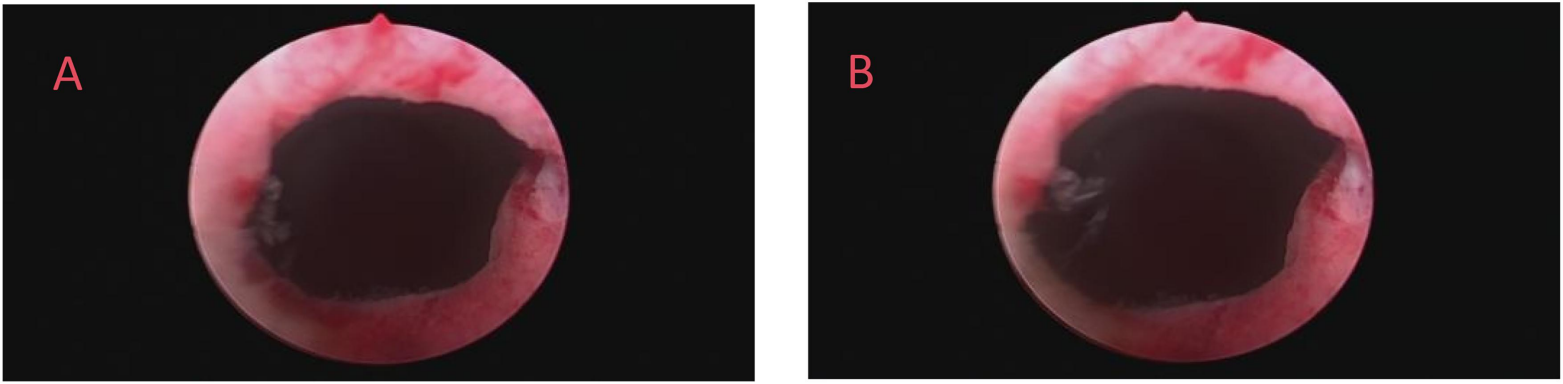
**(A,B)** A cystoscopic evaluation demonstrating satisfactory mucosal healing at the neobladder–urethral anastomosis site in the observation group.

An immunological assessment revealed significantly higher postoperative levels of CD3+ (57.63 ± 3.41% vs. 53.68 ± 3.05%, *p* < 0.01), CD4+ (28.43 ± 2.66% vs. 24.56 ± 2.53%, *p* < 0.01), and the CD4+/CD8+ ratio (0.98 ± 0.15 vs. 0.83 ± 0.09, *p* < 0.01) in the observation group, while CD8+ levels remained comparable between the groups (29.30 ± 3.04% vs. 29.72 ± 1.43%, *p* > 0.05). Both groups exhibited significant postoperative decreases in all immune parameters compared with preoperative levels (*p* < 0.01) (Table 4). Complication rates were similar between the groups, with the observation group experiencing five infections, three gastrointestinal complications, and two urinary fistulas versus three infections, two gastrointestinal complications, and four fistulas in the control group (*p* > 0.05). No differences were noted in catheter duration or time to return of bowel function (*p* > 0.05) (Table 2). A cystoscopic follow-up confirmed satisfactory mucosal healing at the anastomotic site in the observation group (Figure 2). Regarding anastomotic stricture, no patient in either group developed a clinically significant urethral or neobladder-neck stricture that required endoscopic or surgical intervention during the follow-up period. In the observation group, one patient (3.3%) exhibited mild, asymptomatic narrowing without clinical symptoms, while in the control group, two patients (8.0%) had mild asymptomatic narrowing. However, these differences were not statistically significant (*p* > 0.05).

**Table 4:** Patient immune function indicators.

Group	Time	CD3%	CD4%	CD8%	CD4/CD8%
Observation group (*n* = 30)	Preoperative	59.8 ± 2.68	31.53 ± 2.01	26.73 ± 1.36	1.18 ± 0.10
	1 week postoperatively	57.63 ± 3.41^b^	28.43 ± 2.66^b^	29.30 ± 3.04^b^	0.98 ± 0.15^b^
Control group (*n* = 25)	Preoperatively	60.6 ± 3.11	30.44 ± 2.90	26.48 ± 1.68	1.15 ± 0.13
	1 week postoperatively	53.68 ± 3.05^a^^,^^b^	24.56 ± 2.53^a^^,^^b^	29.72 ± 1.43^b^	0.83 ± 0.09^a^^,^^b^

Compared with the control group ^a^*p* < 0.01; compared with preoperative values ^b^*p* < 0.01.

## Discussion

Laparoscopic radical cystectomy, regarded as the standard treatment for muscle-invasive bladder cancer, plays a crucial role in improving patient prognosis because of its minimally invasive advantages (15). With a 5–10× magnified visual field, this technique allows for clear identification of key anatomical structures, such as the pelvic neurovascular bundles and seminal vesicles, enabling precise tumor resection and lymph node dissection. It not only significantly reduces intraoperative blood loss and transfusion requirements but also lowers the risk of complications such as rectal injury (16–18).

Ileal orthotopic neobladder reconstruction currently represents the gold standard for urinary diversion after radical cystectomy. By creating a low-pressure reservoir that simulates physiological voiding, it alleviates the psychological burden and lifestyle inconveniences associated with an abdominal stoma (19, 20). Reconstruction of the neobladder and urethra is essential for patient recovery (21). However, traditional interrupted suture techniques have notable limitations in neobladder–urethral anastomosis (22). Within the confined laparoscopic operative space, restricted needle-holder angles make suturing challenging; sutures tend to entangle during knot tying, and uneven tension can lead to anastomotic ischemia (23, 24). Although robotic surgery offers superior instrument articulation, its high cost limits widespread use in primary hospitals (25).

To overcome these technical challenges, researchers worldwide have explored various innovative approaches in recent years (26, 27). Hu et al. employed a modified U-shaped ileal neobladder to facilitate the neobladder–urethral anastomosis by minimizing tension at the anastomotic site, although this method did not fully address the difficulty of urethral suturing (28). Ficarra et al. reconstructed the neobladder–urethra using a novel urethral fixation technique that significantly improved daytime and nocturnal urinary continence without increasing perioperative complications, although further studies on operative duration and postoperative immune status were not conducted (29). While these techniques have advanced urethrovesical anastomosis and urinary continence, limitations remain because of the complex pelvic anatomy, with persistent challenges in deep-space visualization, anastomotic precision, and long-term functional outcomes (30, 31).

To address the limitations of traditional suturing techniques in neobladder–urethral anastomosis, we innovatively developed a technique called urethral dragging-and-adhesion anastomosis. This novel, suture-free approach utilizes external traction to precisely draw the urethral stump into the neobladder neck, enabling tension-free anastomosis through the natural regenerative capacities of transitional and glandular epithelia (32). After LRC and pelvic lymph node dissection, the specimen is extracted through a small incision, and IONB reconstruction with urethral anastomosis is performed extracorporeally. This breakthrough technique allows for precise visualization-controlled regulation of the anastomotic margins and suture spacing, significantly reducing intraoperative technical difficulty and the risk of postoperative complications such as urinary leakage and anastomotic stricture. It not only markedly shortens operative times (279.80 ± 45.32 min vs. 382.60 ± 45.94 min, *p* < 0.01) and vesicourethral anastomosis times (18.37 ± 7.05 min vs. 61.80 ± 11.35 min, *p* < 0.01) but also results in less blood loss (274.00 ± 85.48 mL vs. 336.80 ± 72.44 mL, *p* < 0.01). Furthermore, there were no significant differences in immediate postoperative urinary control or urodynamic parameters between this technique and traditional suturing methods. However, in the observation group utilizing the urethral dragging-and-adhesion anastomosis method, postoperative levels of CD3+ immune cells (57.63 ± 3.41% vs. 53.68 ± 3.05%, *p* < 0.01), CD4+ immune cells (28.43 ± 2.66% vs. 24.56 ± 2.53%, *p* < 0.01), and the CD4+/CD8+ ratio (0.98 ± 0.15 vs. 0.83 ± 0.09, *p* < 0.01) were significantly higher than those in the control group. No significant difference was observed in CD8+ immune cell levels between the two groups (29.30 ± 3.04% vs. 29.72 ± 1.43%, *p* > 0.05). In addition, both groups exhibited significant postoperative reductions in CD3+, CD4+, and CD8+ immune cell counts and the CD4+/CD8+ ratio compared with preoperative levels (*p* < 0.01). The observed immunomodulatory effects arise from precise mucosal approximation, which minimizes ischemia; reduced urethral stump exposure, which limits bacterial colonization; and optimized anastomotic tension, which prevents tissue microtrauma (33). This surgical stress response modulation may improve prognosis by preserving antitumor immunity, reducing sepsis risk, and maintaining immune homeostasis. These findings demonstrate that refined anastomotic techniques not only enhance anatomical reconstruction but also provide immunological benefits extending beyond the postoperative period, potentially improving long-term oncologic outcomes (34). The ability to favorably influence surgical immune responses through technical optimization represents a critical advancement in oncologic surgery, where immune status significantly impacts survival.

Several important limitations of this study should be acknowledged. Although this was a dual-center investigation, the sample size remained relatively modest, and no *a priori* power calculation was performed. As a retrospective pilot study, the sample was determined by consecutive eligibility during predefined study periods, which limits statistical power and increases the risk of type II error. Clinically meaningful differences may therefore not have been detected, and the findings should be regarded as only preliminary and hypothesis-generating. The chronological allocation of patients, reflecting the gradual adoption of the modified technique, introduces potential selection bias and learning curve effects. Although all procedures were performed by experienced surgeons who had undergone structured training, the delayed timing of the modified technique group may have contributed to shorter operative times and reduced blood loss. As such, causal inference is limited, and the observed outcomes could not be definitively attributed to the anastomotic technique alone. The study population consisted entirely of male patients, which represents a significant limitation. Anatomical differences between men and women, particularly in pelvic anatomy and urethral support structures, may influence operative procedures and surgical outcomes, and therefore, our findings may not be directly generalizable to female populations. In addition, although the participating surgeons at our centers may have less cumulative experience with female orthotopic neobladder reconstruction, this may not necessarily reflect national trends, as specialized tertiary centers often have surgeons proficient in procedures on both male and female patients. Furthermore, all patients were of Han Chinese origin, which provides important demographic context but also highlights the need for validation of these findings in more diverse and multiethnic populations. Future prospective studies incorporating female cohorts and broader demographic representation are warranted to confirm the efficacy and safety of this technique in different patient populations.

With respect to immune assessment, markers were evaluated at only two time points—preoperatively and at 1 week postoperatively. No long-term immunological data were collected, and claims regarding sustained immune preservation were not supported by the evidence at hand. Moreover, the observed differences in immune parameters, although statistically significant, were modest in magnitude (e.g., CD4+/CD8+ ratio 0.98 ± 0.15 vs. 0.83 ± 0.09), and their clinical relevance remains uncertain. Whether such differences translate into meaningful reductions in postoperative infection or improved oncological outcomes has yet to be demonstrated. Although continence and urodynamic parameters were clearly defined and rigorously assessed, long-term functional outcomes—including durable continence and comprehensive urodynamic evaluation—require further investigation. Important oncological endpoints such as recurrence-free and tumor-specific survival were not systematically evaluated in this study, as the median follow-up period of 24 months remains relatively limited for conducting a mature survival analysis. Quality-of-life outcomes and patient-reported measures were also not incorporated. In addition, although the modified technique was described in detail to enhance reproducibility, standardized endoscopic images of the traditional anastomosis were not available for comparative purposes, which limited the visual evidence supporting our technical claims.

Several methodological aspects warrant further discussion. First, the observed reductions in operative time and urethral anastomosis time may be partially attributable to a learning-curve effect, as the modified technique was introduced only after the conventional method. The accumulating experience of the surgical team over time could have contributed to improved efficiency; therefore, a prospective randomized trial is necessary to distinguish the true effect of the technique from the influence of the learning curve. Second, in many high-volume centers, robot-assisted radical cystectomy with intracorporeal urinary diversion has become the standard of care. However, in resource-limited settings where robotic platforms are not readily accessible, conventional laparoscopic surgery remains a valuable and cost-effective alternative. The modified urethral traction and adhesive anastomosis technique described herein offers a feasible and effective option for these regions. Future studies should validate our findings in robot-assisted cases to enhance the generalizability of this technique. Third, regarding long-term outcomes, no clinically significant anastomotic strictures requiring intervention were observed during follow-up. Mild, asymptomatic strictures occurred in 3.3% of patients in the observation group and 8.0% in the control group, with no statistically significant difference between the two groups. These low incidence rates suggest that the modified technique does not increase the risk of stricture formation compared to the conventional method. Nevertheless, extended follow-up is needed to fully assess the incidence of late-onset strictures.

## Conclusion

The urethral dragging-and-adhesion anastomosis technique may contribute to improved surgical efficiency and immune function in neobladder reconstruction following laparoscopic cystectomy. It appears to maintain urinary control outcomes and safety profiles comparable to those of traditional procedures. This technique may therefore hold certain clinical value and could represent a promising modification worthy of further investigation and broader clinical validation.

## Data Availability

The raw data supporting the conclusions of this article will be made available by the authors, without undue reservation.
